# Drug consumption in German cities and municipalities during the COVID-19 lockdown: a wastewater analysis

**DOI:** 10.1007/s00210-022-02377-2

**Published:** 2023-01-12

**Authors:** Reinhard Oertel, Sara Schubert, Björn Helm, Robin Mayer, Roger Dumke, Ali El-Armouche, Bertold Renner

**Affiliations:** 1 Institute of Clinical Pharmacology, Faculty of Medicine Carl Gustav Carus, Technische Universität Dresden, Fetscherstrasse 74, 01307 Dresden, Germany; 2 Institute of Hydrobiology, Technische Universität Dresden, Helmholzstrasse 10, 01069 Dresden, Germany; 3 Institute of Urban and Industrial Water Management, Technische Universität Dresden, Helmholtzstrasse 10, 01069 Dresden, Germany; 4 Institute of Medical Microbiology and Virology, University Hospital Carl Gustav Carus, Technische Universität Dresden, Fetscherstrasse 74, 01307 Dresden, Germany; 5 Institute of Pharmacology and Toxicology, Faculty of Medicine Carl Gustav Carus, Technische Universität Dresden, Fetscherstrasse 74, 01307 Dresden, Germany

**Keywords:** Illicit drugs, Cotinine, Wastewater-based epidemiology, Marker substances, SARS-CoV-2

## Abstract

Analysis of illicit drugs, medicines, and pathogens in wastewater is a powerful tool for epidemiological studies to monitor public health trends. The aims of this study were to (i) assess spatial and temporal trends of population-normalized mass loads of illicit drugs and nicotine in raw wastewater in the time of regulations against SARS-CoV-2 infections (2020–21) and (ii) find substances that are feasible markers for characterizing the occurrence of selected drugs in wastewater. Raw sewage 24-h composite samples were collected in catchment areas of 15 wastewater treatment plants (WWTPs) in urban, small-town, and rural areas in Germany during different lockdown phases from April 2020 to December 2021. Parent substances (amphetamine, methamphetamine, MDMA, carbamazepine, gabapentin, and metoprolol) and the metabolites of cocaine (benzoylecgonine) and nicotine (cotinine) were measured. The daily discharge of WWTP influents were used to calculate the daily load (mg/day) normalized by population equivalents (PE) in drained catchment areas (in mg/1,000 persons/day). A weekend trend for illicit drugs was visible with higher amounts on Saturdays and Sundays in larger WWTPs. An influence of the regulations to reduce SARS-CoV-2 infections such as contact bans and border closures on drug consumption has been proven in some cases and refuted in several. In addition, metoprolol and cotinine were found to be suitable as marker substances for the characterization of wastewater. A change in drug use was visible at the beginning of the SARS-CoV-2 crisis. Thereafter from mid-2020, no obvious effect was detected with regard to the regulations against SARS-CoV-2 infections on concentration of drugs in wastewater. Wastewater-based epidemiology is suitable for showing changes in drug consumption during the COVID-19 lockdown.

**Supplementary Information:**

The online version contains supplementary material available at 10.1007/s00210-022-02377-2.

## Introduction

Investigations of water are carried out primarily for toxicological reasons, especially in the case of drinking water and surface water. In the case of wastewater, different studies performed (eco-)toxicological investigations in the inflow and outflow of wastewater treatment plants (WWTPs), for example, on hormones (estrogenic activity) and occurrence of drug-resistant bacteria (Rossmann et al. 2014; Marx et al. 2015; Kaeseberg et al. 2018).

There are already numerous approaches using wastewater for epidemiological studies indicating that wastewater-based epidemiology (WBE) could become a powerful tool for monitoring public health trends through the analysis of biomarkers such as drugs, chemicals, and pathogens (Castiglioni et al. 2013; Prichard et al. 2014; Gurke et al. 2015; Mao et al. 2018). Over the last decade, the application of WBE to monitor illicit drug loads increased. As there is a limited number of high-quality studies, further standardization of the WBE approach for illicit drugs is needed especially with regard to the sampling methodology (Huizer et al. 2021). Examples are the cross-European drug screening carried out since 2011 by the network Sewage Analysis Core Group Europe (SCORE) and the European Monitoring Center for Drugs and Drug Addiction (EMCDDA) (Gonzalez-Marino et al. 2020).

The current activities to establish an early warning system for SARS-CoV-2 epidemic peaks has brought WBE strongly into focus (Erickson et al. 2021; Price et al. 2022, Wainwright et al. 2020; Bade et al. 2021; Been et al. 2021; Cisneros and Cunningham 2021; Manchikanti et al. 2021; Palamar et al. 2021; Reinstadler et al. 2021; Helm et al. 2022). The study presented here deals with characterization of changes in the consumption patterns of illicit drugs and nicotine in a project for SARS-CoV-2-tracking in Germany (Helm et al. 2022). It has been hypothesized that physical distancing and social limitations disproportionately affect people who regularly use illegal drugs and could lead to shifts in the illegal drug markets (Dietze and Peacock 2020; Bade et al. 2021). In the Global Financial Crisis 2008, compound specific changes of illicit drug consumption were observed. The use of expensive drugs, such as cocaine, decreased, and the use of cheaper synthetic drugs, such as amphetamine, increased (Dom et al. 2016; Thomaidis et al. 2016). Recently, several studies from Innsbruck, Austria (Reinstadler et al. 2021), selected European cities (Been et al. 2021), Australia (Price et al. 2022; Bade et al. 2021), Italy (Di Marcantonio et al. 2022), Greece (Alygizakis et al. 2021), Reykjavik, Iceland (Love et al. 2022), and the USA (Erickson et al. 2021; Cisneros and Cunningham 2021; Manchikanti et al. 2021; Palamar et al. 2021) on the influence of measurements to reduce SARS-CoV-2 infections on drug consumption showed no clear trends. So far, there have only been some studies in Germany that have surveyed consumers about the effects of the lockdown on drug use. The impact was relatively small (Scherbaum et al. 2021; Pavarin et al. 2022).

A second aspect of these investigations are methodological improvements of the WBE. In previous studies, it could be shown that there is a correlation between the amount of prescription drugs and their recovery in wastewater (Cisneros and Cunningham 2021). However, the significance of this correlation is depending on various prerequisites, mainly regarding the wastewater sampled and the drug investigated. First of all, the wastewater samples should be representative for sewage flow of the population considered. Therefore, 24-h composite samples with information about the flow rate are standard procedures. In practice, the technical requirements for this sampling are not always given, for example, in the case of random samples from the sewer network or during heavy rain events (Castiglioni et al. 2013; Huizer et al. 2021). If only the concentrations are known in these cases, but not the flow rates, the amounts might be estimated using suitable and validated target substances.

Our study is aimed at determining the influence of COVID-19 and the related restrictions on drug consumption in Germany with wastewater-based epidemiology.

## Method

### Wastewater samples

**Fig. 1:**
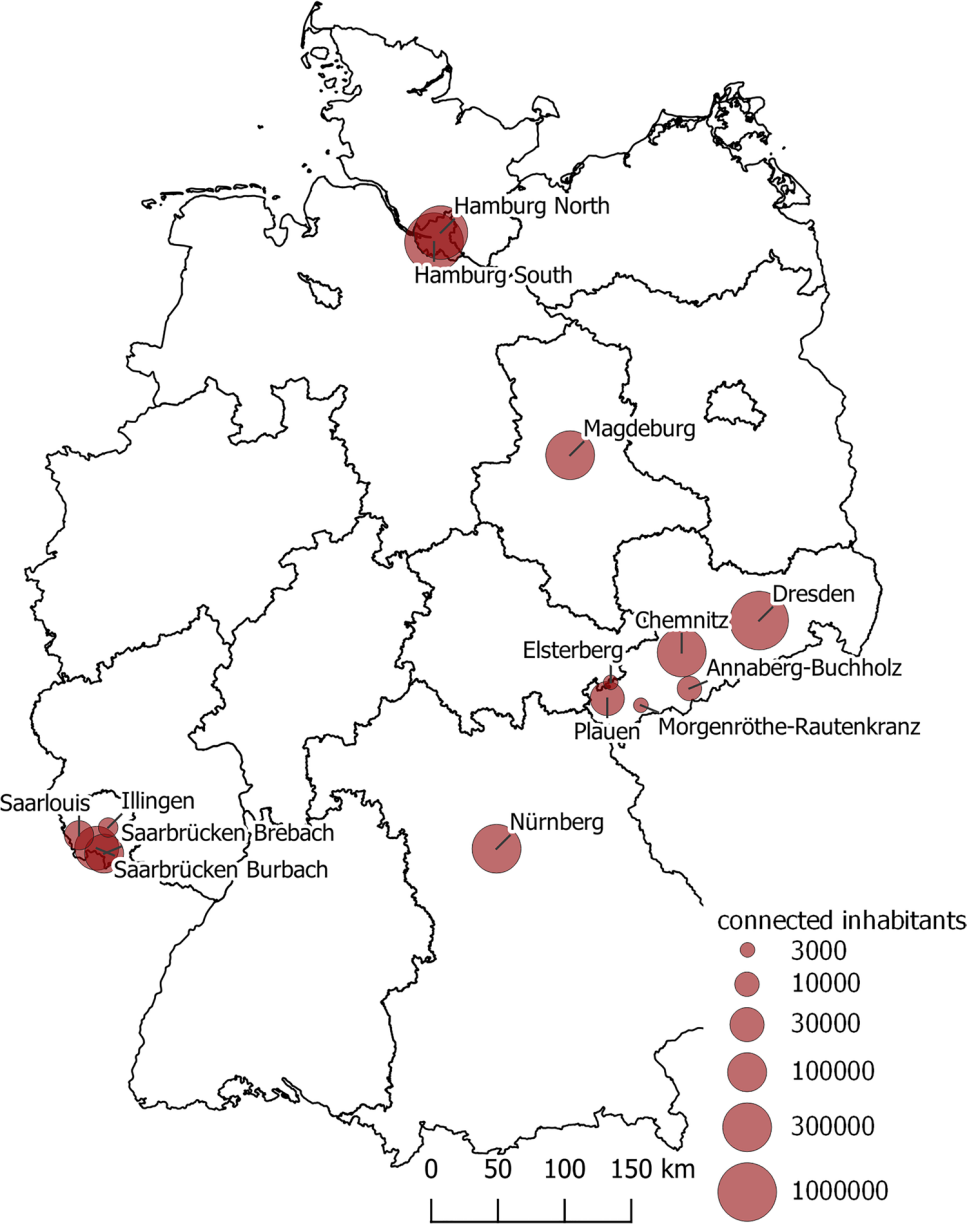
Spatial distribution of sampling sites in Germany and number of inhabitants connected to the wastewater treatment plants; site MLHC is not shown due to anonymity requirements

The study included about 800 24-h composite wastewater samples collected at the influents of 15 German WWTPs differing in size-classes (in population equivalent PE; Table 1). WWTPs of Dresden, Chemnitz, Plauen, Annaberg-Buchholz, Elsterberg, Morgenröthe-Rautenkranz, and a municipality with a large health clinic (MLHC) were sampled between April 2020 and December 2021 and WWTPs of Hamburg (Hamburg North, Hamburg South), Magdeburg, Nuremberg, and Saarland (Saarbr. Brebach, Saarbr. Brubach, Saarlouis, Wustweiler) between April 2020 and July 2020 (Fig. 1). Typically, all 24-h composite samples were collected at the WWTP influent from 8 a.m. to 8 a.m., therefore mainly representing a composite sample of the day when sampling started. In the most cases, wastewater was automatically sampled in flow proportional way (WWTP of Dresden, Chemnitz, Plauen, Annaberg-Buchholz, Hamburg, Magdeburg, Nuremberg, and Saarland) only in the very small WWTPs in time proportional way (MLHC, Elsterberg, and Morgenröthe-Rautenkranz). Wastewater samples were frozen at – 18 °C and then sent collectively in polystyrene boxes with frozen thermal bags within a maximum of 24 h to ensure the frozen state of samples before arriving the lab.

**Table 1:** Inhabitant specific daily loads of pharmaceuticals in the influent of different German WWTP

Sampling period	WWTP	Residential population	Number of samples
Jan20–Dec2021	Annaberg-Buchholz	29.400	123
Jan20–Dec2021	Chemnitz	239.402	151
Jan20–Dec2021	Dresden	671.806	226
2020	Hamburg Nord	630.000	25
2020	Hamburg Süd	1.420.000	26
2020	Nuremberg	1.038.704	32
2020	Magdeburg	278.236	42
2020	Illingen Wustweiler	27.069	20
2020	Saarbrücken Brebach	80.637	20
2020	Saarbrücken Burbach	123.006	19
2020	Saarlouis	43.467	20
Jan20–Dec2021	Plauen	64.597	112
Jan21–June2021	MLHC	3.568	3
Jan21–June2021	Elsterberg	3.925	18
Jan21–June2021	MRK	3.025	23

*WWTP*, wastewater treatment plants; *MLHC*, municipality with a large health clinic; *MRK*, Morgenröthe-Rautenkranz

### Selection of targeted compounds

The study included analysis of markers for tobacco and illicit drug use as well as determination of frequently prescribed drugs. The selected prescription medicaments are not expected to be affected by the SARS-CoV-2 restrictions or other seasonal fluctuations (Ludwig et al. 2021) (unpublished prescription data from statutory health insurance AOK Plus, in cooperation with AOK Plus, U. Maywald, Saxony, Germany). The drugs include the beta adrenoceptor antagonist metoprolol, the anticonvulsants carbamazepine and gabapentin, and the antifungal fluconazole.

### Chemical analytics

The nicotine metabolite cotinine and the illegal drugs methamphetamine, amphetamine, MDMA, cocaine, and its metabolite benzoylecgonine as well as the heroin metabolite 6-acetylmorphine were determined as in the SCORE drug screening (Gonzalez-Marino et al. 2020). Analysis were performed with a semi-automatic solid phase extraction (SPE) method and using liquid chromatography coupled to tandem mass spectrometry (LC–MS/MS). The separation was carried out using reversed phase chromatography and tandem mass spectrometry based on (Rossmann et al. 2014; Gurke et al. 2015) using isotope labeled standards. The quantification limits defined as the lowest point of the standard curve was 20 ng/L for the illicit drugs and carbamazepine and 100 ng/L for cotinine, metoprolol, and gabapentin. The acceptance criteria were a signal-to-noise ratio greater than 10 and the intraday and between-day precision had to be lower than 20% deviation.

### Data analysis

The evaluation in part 1 was carried out in accordance with the international standard (Castiglioni et al. 2013). Measured substance concentrations in the wastewater had to be normalized. The daily wastewater flow was known for most of the 24-h composite samples examined. In these cases, the flow rate was used to calculate the load, i.e., the amount of substance that reaches the sewage treatment plant. Additionally mass loads were normalized to the number of people served by the WWTP. The result represents the amount of drug excreted daily per 1000 inhabitants. This value is valid for the comparison between different locations and between different time periods. In part 2 of the study, an alternative normalization with marker substances was tested in order to validate this procedure of a drug related normalization.

## Results

### Study part 1—determination of illicit drugs

For the investigations regarding the influence of the SARS-CoV-2 measures, the available flow rates were used for normalization, and the marker substances were only used for scaling in a few cases. When looking at drug consumption behavior depending on the SARS-CoV-2 measurements, different time periods were set characterizing the extent of restrictions. The investigated samples were assigned to these periods (Table 2). The number of samples per sewage treatment plant and period was different and is given in the Appendix Table 7. For example, there is only one sample for Dresden in the first hard lockdown in April 2020. In other cities, the periods April 2020 and May 2020 up to the opening of the border in June 2020 can be evaluated and compared separately. Unfortunately, there are no samples from WWTPs outside Saxony in 2021.

**Table 2:** Periods of restrictions and regulations due to the SARS-CoV-2 in Germany

Period	Designation	Comment
1 April–30 April 2020	Lockdown 1a (LD1a)	Strict lockdown
1 May–15 June 2020	Lockdown 1b (LD1b)	Reduction of the restrictions
16 June–31 Oct. 2020	Post Lockdown 1 (pLD1)	Border opening, only some restrictions
1 Nov.–15 Dec. 2020	Lockdown light 2 (LDli2)	Restrictions on leisure areas/activities
16 Dec.–28 Feb. 2021	Lockdown 2a (LD2a)	Strict lockdown
1 March–31 May 2021	Lockdown 2b (LD2b)	Lockdown with regional regulations
1 June–22 Nov. 2021	Post Lockdown 2 (pLD2)	Few restrictions
23 Nov. 2021–2 Jan. 2022	Lockdown light 3 (LDli3)	Lockdown with regional regulations

**Fig. 2:**
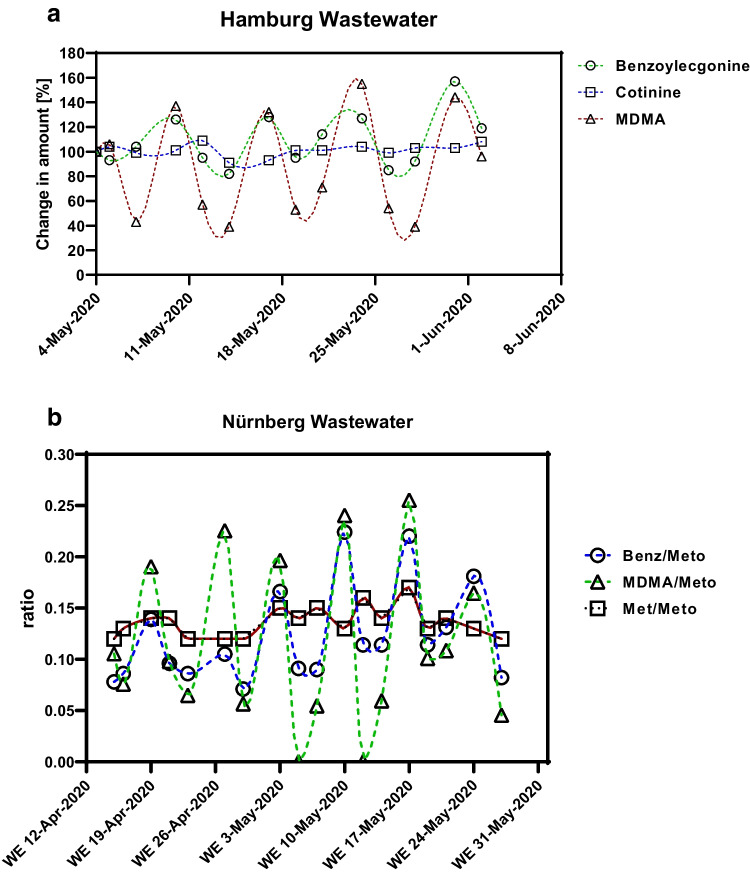
**a** Weekly relative fluctuations in the measured drug concentrations in raw wastewater of Hamburg South, Germany (the value from May 4, 2020 = 100%: benzoylecgonine 449, cotinine 1186, and MDMA 22.1, each value in mg per day and 1000 inhabitants). **b** The amount of benzoylecgonine, MDMA, and methamphetamine detected in the raw water of Nuremberg WWTP (scaled with metoprolol; WE = weekend)

As in previous years’ screening samples, the heroin metabolite 6-acetylmorphine could not reliably be detected in the German WWTPs investigated in the present study. Regional differences in drug use were confirmed for the other illegal drugs. In this study and in previous series of studies, a so-called “weekend trend” was found for certain substances. The “party drug” ecstasy (MDMA) and often cocaine and its metabolite benzoylecgonine were found in larger quantities on weekends than during the week. This has not been established for methamphetamine. Regarding the investigation of an influence of the measurements for SARS-CoV-2 management (contact restriction, border closure) on the change of leisure behavior and of party scene as well on the absolute consumption volumes of drugs, the number of available positive data is a critical point. In some of the smaller Saxon WWTPs, neither ecstasy nor cocaine was detected in most of the samples. Therefore, evaluation was limited to the larger towns. Results from large cities like Hamburg (Fig. 2a) and Nuremberg (Fig. 2b) are shown here as examples.

Samples from Nuremberg and Magdeburg were available for a longer period in spring 2020. In mid-April and from the beginning of June 2020, no samples from the weekend were available, and therefore, no weekend trend was detectable. Nevertheless, the maximum values on the weekends in April 2020 were significantly lower than in May (ratio benzoylecgonine/metoprolol: *p* = 0.029; Mann–Whitney *U*-test), and the concentrations on the working days in May and June 2020 were significantly higher than in April (ratio benzoylecgonine/metoprolol: *p* < 0.001, ratio benzoylecgonine/cotinine: *p* = 0.008; Mann–Whitney *U*-test). Thus, the first lockdown had obviously a strong impact on cocaine consumption (Fig. 2b).

The methamphetamine levels found in Nuremberg were constant over the entire period and independent of the day of the week. In April, the values were 16% higher than in June and July. The party drug MDMA was detected in larger quantities on the weekend than during the weekdays (Fig. 2b). Unfortunately, the samples from the weekends in June and July were not available due to technical reasons.

**Fig. 3:**
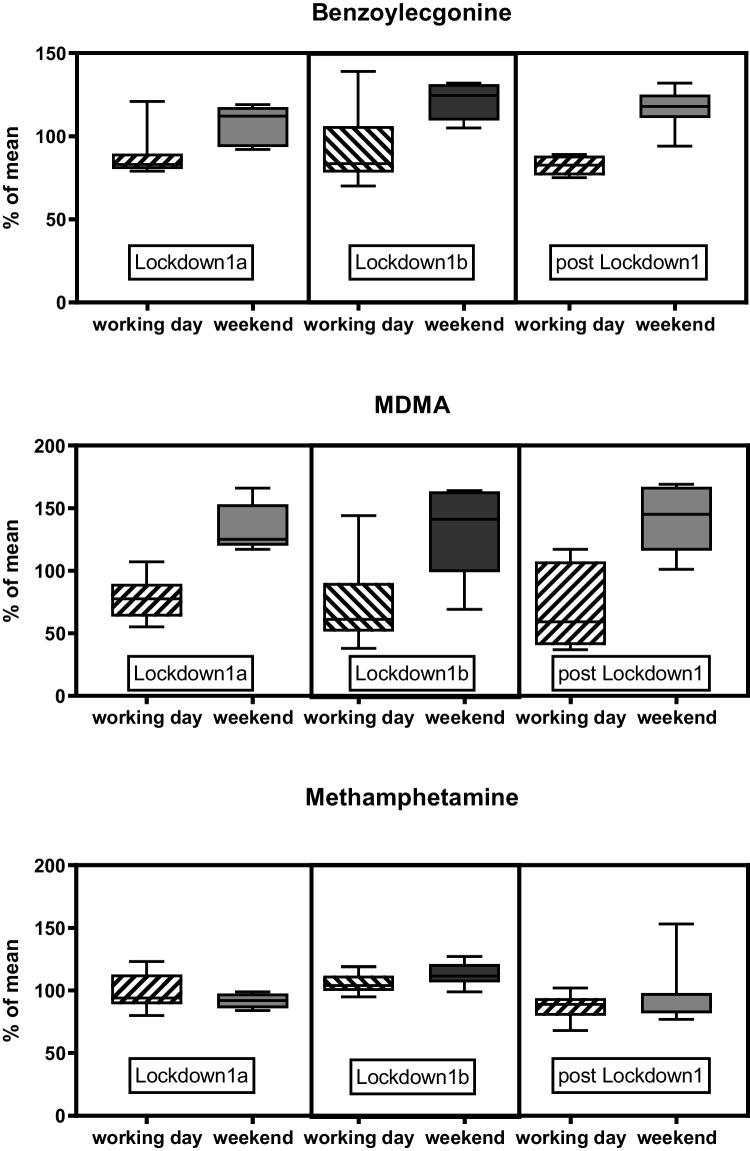
The weekend trend of illicit drugs in raw wastewater of Magdeburg in different periods in 2020 (100% is the mean of all samples from Magdeburg). Horizontal boxlines are showing 25, 50, and 75 percentiles; whiskers represent Min and Max values

The weekend trend of benzoylecgonine, MDMA, and methamphetamine in Magdeburg in Lockdown1 and post Lockdown1 is shown in Fig. 3. Five to ten samples are included in the presented mean values.

Four WWTPs were sampled from the federal state Saarland. Results from the pre-lockdown period were available for two of the WWTPs in 2019. These results can be found in Table 7 in the Appendix. In this area, there is the already known local peculiarity that amphetamine is the drug most consumed in the catchment area and that methamphetamine plays no role up to now. Amphetamine consumption decreased during the lockdown phase and increases again from June 2020 (Appendix Table 8). Consumption did not depend on the day of the week. No clear changes could be identified for cocaine as a result of the regulations according to the SARS-CoV-2 epidemiology. In two WWTPs, MDMA was found in higher concentrations in 2020 than in 2019. As at the other locations examined, MDMA and cocaine are consumed somewhat more on the weekends than during the week. In the time before emergence of SARS-CoV-2, the weekend trend was even more pronounced, especially with MDMA, i.e., during this time only little amount of MDMA was consumed on weekdays.

The most samples of this study were analyzed from the Saxon WWTPs in Dresden and Chemnitz. Further samples come from the WWTPs in Plauen and Annaberg-Buchholz, but samples were not taken at these sites before 2021. In addition, a limited number of results are available from the small wastewater treatment plants in Elsterberg and Morgenröthe-Rautenkranz in 2021. The measured values scattered very widely and, moreover, mostly low concentrations or no drugs of interest were found (Table 3). In contrast to the other WWTPs, the wastewater in these very small WWTPs was sampled in a time-proportional manner.

**Table 3:** Daily excretion of illegal drugs in mg/day per 1000 inhabitants in cities of different sizes in Saxony and, for comparison, cotinine and metoprolol (sampling period April 2020 to June 2021)

	Population equivalent 1000 inhab	*N*	Amphetamine	Benzoylecgonine	MDMA	Methamphetamine	Cotinine	Metoprolol
Chemnitz	239.4	46	15.2	30.0	8.0	255.3	715.4	622.7
Dresden	671.8	140	23.3	52.0	12.9	192.4	699.1	869.0
Plauen	64.6	38	10.0	8.6	1.2	203.4	712.2	650.6
Annaberg-Buchholz	29,4	42	2.5	0.1	1.3	86.1	843.5	621.4
Elsterberg	3.9	18	1.6	0.6	1.6	29.3	372.0	757.5
Morgenröthe-Rautenkranz	3.0	23	2.1	0.5	0.4	4.4	286.3	772.9

With regard to the capacity of the monitored WWTPs, there was a clear positive correlation between population equivalents and the amount of drugs found in wastewater indicating a higher consumption in larger cities. This effect was observed for illegal drugs but this was not true for cotinine and metoprolol: Spearman correlation amphetamine (*r* = 0.943; *p* = 0.005), benzoylecgonine (*r* = 0.829; *p* = 0.042), MDMA (*r* = 1; *p* < 0.001), methamphetamine (*r* = 0.943; *p* = 0.005), cotinine (*r* = 0.6 n.s.), and metoprolol (*r* =  − 0.086 n.s.).

In Dresden, Chemnitz, Plauen, and Annaberg-Buchholz, as in the locations already described, relatively constant amounts of methamphetamine, amphetamine, and cotinine were found throughout the week. For MDMA and cocaine, the consumption on the weekend was significantly higher than during the week and that over the entire period of the study (Mann–Whitney *U*-test: *p* < 0.001). This pattern was independent from restrictions to reduce SARS-CoV-2 infections. From the two turns of the year 2020/21 and 2021/22, four samples from the WWTP Dresden were available. Strict anti-SARS-CoV-2 rules applied during these periods. We found extremely high amounts of MDMA and cocaine/benzoylecgonine used as party drugs indicate that the SARS-CoV-2 regulations on New Year’s Eve were not fully implemented. Results in Table 4 show a weekend trend of increased use of cocaine and MDMA and the even higher drug use at New Year’s Eve in Dresden (Kruskal–Wallis-Test: *p* < 0.001; New Year vs. weekend: MDMA *p* < 0.001, cocaine/benzoylecgonine *p* > 0.05 (ns); New Year vs. week: MDMA *p* < 0.001, cocaine/benzoylecgonine *p* = 0.006).

**Table 4:** Mean daily amounts of benzoylecgonine and MDMA found in the raw water of Dresden daily from 1 April 2020 to 1 January 2022

		Benzoylecgonine	Benzoylecgonine	MDMA	MDMA
	*N*	g/day	%^1^	g/day	%^1^
All samples	226	30.9	100%	8.4	100%
Weekend	69	40.5	131%	13.8	163%
Workdays	153	26.5	86%	6.0	71%
New Year’s Eve	4	68.7	222%	34.9	415%

^1^Means of weekend, workdays, and New Year’s Eve are normalized to mean of all samples

**Fig. 4:**
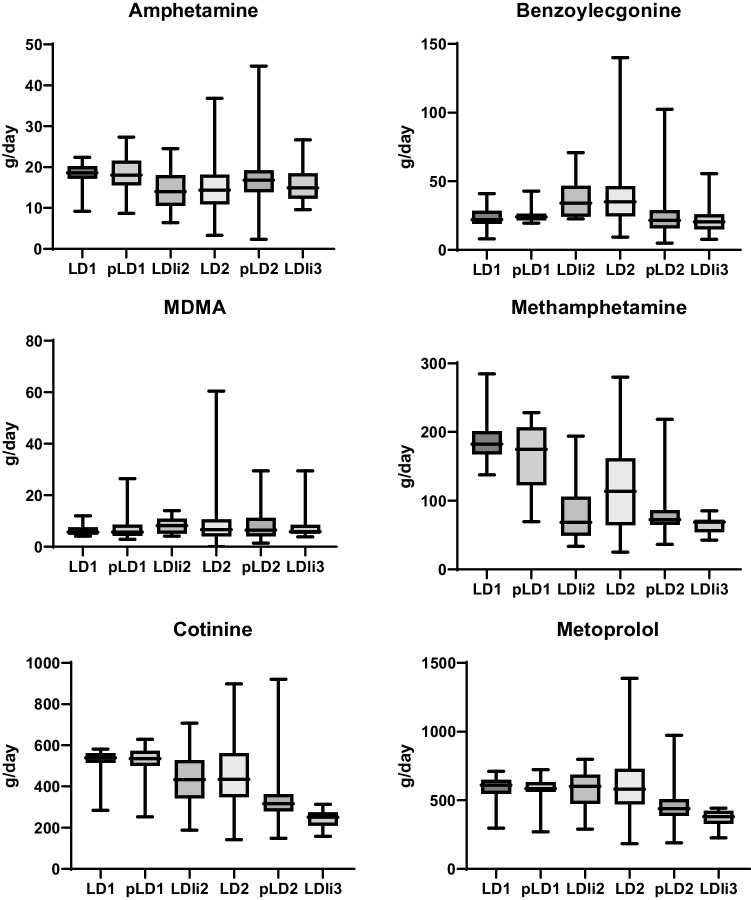
Amount of illicit drugs, cotinine, and metoprolol in wastewater of Dresden depending on SARS-CoV-2 regulations. Whisker boxplots are showing median, 25 and 75% percentile, and Min and Max values. Values represent amounts calculated from flow rates (abbreviations of the restriction periods are explained in Table 2)

Figure 4 shows changes in drug consumption: in the first lockdown phase (LD1 and pLD1), methamphetamine and to a lesser extend its metabolic product amphetamine were detected in higher concentrations in comparison with those in the rest of the study period and before restrictions against SARS-CoV-2 (Gonzalez-Marino et al. 2020). Cocaine metabolite have been found in larger quantities since autumn 2020 (LDli2) and also in the period before SARS-CoV-2 (Gonzalez-Marino et al. 2020). MDMA was found in a lesser extent in the first lockdown phase. On the weekends in the first lockdown period, the measured MDMA amounts were slightly lower than at later periods. Little higher concentrations of cotinine were determined in the first lockdown phase.

**Fig. 5:**
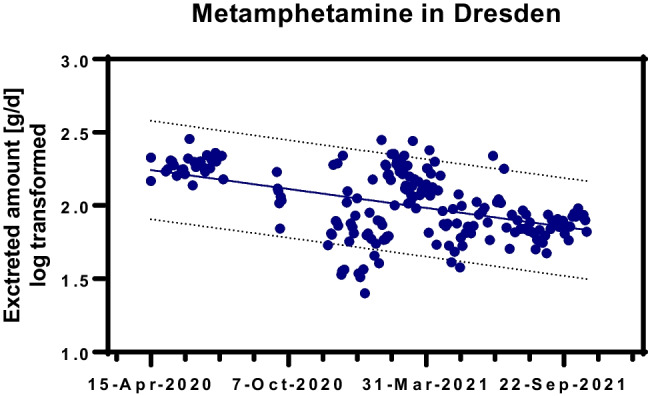
The amount of methamphetamine observed in Dresden from the first lockdown phase in April 2020 to lockdown light 3 at the end of 2021. A significant decrease was observed throughout the sample period (linear regression analysis on log transformed *y* values with GraphPad Prism 9.3.1; best-fit regression line and 90% prediction bands are shown; 95% CI for slope  − 6.55 to  − 3.81 × 10^–3^; *F* = 55.7; *dF* = 1193; *p* < 0.0001)

Figure 5 shows that the amount of methamphetamine excreted in the wastewater of Dresden decreased significantly by the end of 2021 after higher values in the first lockdown phase. The other observed differences in drug use were not significant.

### Study part 2—establishment of marker substances

Sometimes, only the drug concentrations in waste water are known, and the calculation of the amount is not possible, for example, in the case of random samples from the sewer network or in the event of heavy rain events. In these cases, a normalization of the measured concentrations could be desirable. It was tested whether the prescription drugs metoprolol, carbamazepine, gabapentin, and fluconazole are excreted daily in the different locations in the same amounts and whether this amount is comparable per inhabitant of the different catchment areas.

For that purpose, all sample results from 2020 and from the first half of 2021 were evaluated. Data from 2020 and 2021 were available for three sewage treatment plants (Dresden, Chemnitz, and Annaberg-Buchholz). These were first analyzed separately. No obvious difference was observed between the 2 years, so that these values will be summarized for further evaluations.

In contrast to previous investigations, fluconazole could not be detected in numerous samples from smaller sewage treatment plants. Therefore, this parameter was excluded from further analysis. Table 5 shows the number of samples, the mean of the elimination amount, and the coefficient of variation for three active substances and one metabolite in the raw water from 13 plants in Germany over the entire specified period.

**Table 5:** Inhabitant specific daily loads of pharmaceuticals in the influent of different German WWTP

Sampling period	WWTP	Number of samples	Carbamazepine		Gabapentin		Metoprolol		Cotinine	
			mg/day/1000Inhab		mg/day/1000Inhab		mg/day/1000Inhab		mg/day/1000Inhab	
		*N*	Mean	CV	Mean	CV	Mean	CV	Mean	CV
Jan20–June2021	Annaberg-Buchholz	45	211.0	*48%*	3171.5	*32%*	628.4	18%	863.7	16%
Jan20–June2021	Chemnitz	46	136.3	*34%*	2741.3	25%	622.7	19%	715.4	19%
Jan20–June2021	Dresden	138	134.7	*33%*	2256.7	*40%*	869.0	27%	690.5	29%
2020	Hamburg North	25	44.8	22%	649.2	28%	353.9	14%	882.5	16%
2020	Hamburg South	26	75.2	16%	734.2	*31%*	581.6	18%	1202.6	13%
2020	Illingen Wustweiler	20	126.0	28%	1704.9	14%	670.4	10%	771.8	11%
2020	Saarbr. Brebach	20	117.3	19%	1751.2	13%	560.4	14%	888.7	7%
2020	Saarbr. Burbach	19	120.7	*36%*	1831.8	19%	349.9	15%	1188.1	14%
2020	Saarlouis	20	200.3	20%	1903.8	15%	657.8	15%	1155.4	14%
Jan21–June2021	Plauen	38	158.7	*45%*	1623.3	*39%*	575.8	23%	596.1	29%
2020	MLHC	3	171.0	*45%*	3692.7	*43%*	1820.1	2%	1149.5	27%
Jan21–June2021	Elsterberg	18	26.8	*52%*	1564.2	*53%*	757.5	*42%*	372.0	*55%*
Jan21–June2021	Morgenröthe-Rautenkranz	23	173.3	28%	1130.8	*32%*	772.9	22%	286.3	26%

Values in italics indicate a high coefficient of variation (> 30%)

*WWTP*, wastewater treatment plants; *CV*, coefficient of variation is the ratio of the standard deviation to the mean; *MLHC*, municipality with a large health clinic

The assessment of the other substances, which were originally intended to be examined to determine whether the lockdown and other restrictions due to the epidemiology of SARS-CoV-2 resulted in a change in consumer behavior, showed that cotinine was always very easily detectable and the amount excreted per 1000 inhabitants per day was always relatively constant (Table 5 last column). Cotinine is a stable metabolic product of nicotine and thus a marker for tobacco consumption (Shahab et al. 2009). Smoking is still very common, and the SARS-CoV-2 regulations obviously had an insignificant effect on consumption.

**Fig. 6:**
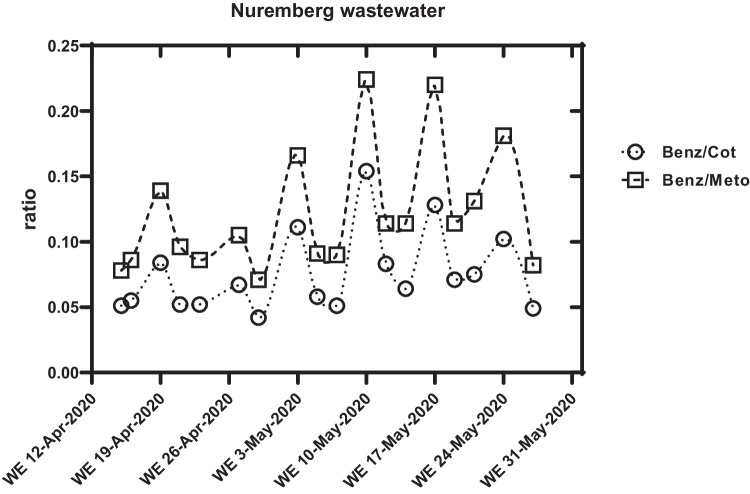
The amount of benzoylecgonine detected in the raw wastewater of Nuremberg, Germany (scaled with cotinine or metoprolol; ratio = quotient of benzoylecgonine concentration divided by marker concentration; WE = weekend)

In this study, we had no information on the water flow rate for the WWTP of Nuremberg and Magdeburg. Therefore, the drug concentrations were scaled with the assumed constant cotinine and metoprolol concentrations. The scaling with cotinine and metoprolol provided very similar, comparable results (Fig. 6). For reasons of clarity, only normalization with metoprolol was shown here, and the concentrations of MDMA and methamphetamine were normalized with metoprolol (Fig. 2b).

## Discussion

### Study part 1—determination of illegal drugs

The hypothesis of this study was that the SARS-CoV-2 regulations, such as contact bans and border closures, would affect illicit drug use. This has been proven in some cases and disproven in several others. Wastewater samples from April 2020 were available from Nuremberg and Magdeburg. In the first lockdown in April 2020, an influence of the regulations on drug use was observed in these samples. The data from Nuremberg and Magdeburg showed that the party drugs were evenly consumed throughout the week in April 2020 without any “weekend trend.” In contrast to that, a “weekend trend” is known from earlier series of studies for the “party drug” ecstasy (MDMA) and cocaine/benzoylecgonine (Gonzalez-Marino et al. 2020; Wainwright et al. 2020). Typically, methamphetamine loads are evenly distributed throughout the week. Thus, like cotinine, methamphetamine shows no weekend trend and was consumed evenly. In May 2020, the “weekend trend” was significantly higher again (Figs. 2b and 3). Also, the results of the samples from the WWTP in Hamburg from May to July 2020 show strong differences depending on the day of the week for appropriate illicit drugs. The maximum and minimum amounts of MDMA and cocaine excreted per inhabitant and day remained relatively constant over the 2 months. An effect of regulations against SARS-CoV-2 infections on the consumption of illegal drugs and parties was not detectable during this period (Fig. 2a).

The results of this study also indicated that the amount of benzoylecgonine in Nuremberg wastewater decreased during the first lockdown. In Innsbruck, an increase in the use of methamphetamine and a decrease in MDMA and cocaine were found in the first lockdown (Manchikanti et al. 2021). In our data, less benzoylecgonine was also found in wastewater in Germany. The other two trends (for methamphetamine and MDMA) could not be shown. The samples from Saarland are of note because there were comparative samples from the time before the SARS-CoV-2 pandemics. Amphetamine use decreased regardless of the day of the week during the lockdown phase and increased again from June 2020. No significant changes were recognizable for cocaine consumption due to the SARS-CoV-2 regulations. MDMA was found significantly more in 2020 than in 2019. MDMA and cocaine were consumed more on the weekends than during the week (Table 7). This is observed in the wastewater of different cities. In the time before emergence of SARS-CoV-2, the weekend trend was even more pronounced, especially for MDMA, i.e., during this time, only little ecstasy was consumed on workdays. Further studies should investigate whether this change in consumer behavior is reversible.

The samples from the Dresden and Chemnitz WWTP offer the opportunity to monitor drug consumption over a longer period from Lockdown 1 to Lockdown light 3. The samples from Plauen and Annaberg-Buchholz as well from the Elsterberg and Morgenröthe-Rautenkranz small plants from 2021 were intended to investigate the difference in drug consumption between urban, small-town, and rural areas. The measured values in the plants treating smaller populations fluctuate strongly, and mostly, no or only low concentrations of drugs were found. The fluctuations could be increased by the time-proportional sampling. Therefore, precise evaluation of drug distribution pattern is more difficult. The results in Table 3 clearly show the different consumer behavior of the urban and rural populations. While the medically prescribed beta adrenoceptor antagonist metoprolol was distributed equally in all wastewaters investigated, we determined strong differences in the concentration of illegal drugs, most notably in cocaine and MDMA, but also significantly in methamphetamine. The differences were so large that any fluctuations between the different periods can be neglected. The size of the city also played a role here. The reasons for this are related to lifestyle, demographics, and social differences.

The amounts of amphetamines occurring in the wastewater in Saxony are very low compared to the amounts of methamphetamine. It can be assumed that most of them were the metabolic product of methamphetamine, and only a small amount was consumed directly. In Dresden, Chemnitz, Plauen, and Annaberg-Buchholz, as in the locations already described, relatively constant amounts of methamphetamine, amphetamine, and cotinine were found during the week. For MDMA and cocaine, the consumption on the weekend was significantly higher than during the week, and this was true for the entire period of the study, i.e., regardless of regulations to control SARS-CoV-2.

Dresden samples from both the turn of the year 2020/21 and 2021/22 represent periods with strict rules against SARS-CoV-2. In wastewater collected during these time periods, more than four times the amount of MDMA and more than twice the amount of cocaine/benzoylecgonine in comparison to the mean of all samples were found indicating that the regulations were not fully respected on these holidays (Table 4).

Looking at the long period from Lockdown 1 to Lockdown light 3, the different changes in consumption can be seen in Dresden. In the first lockdown phase, methamphetamine use was significantly higher than in the rest of the study period and before measurements against SARS-CoV-2. The amount of methamphetamine excreted in Dresden decreased significantly from the first lockdown phase in April 2020 to Lockdown Light 3 at the end of 2021. Cocaine and its metabolite have been found in larger quantities since the summer of 2020 and also before the SARS-CoV-2 pandemics. At this location, higher cocaine consumption has been observed during the second half of 2020, which cannot be explained by SARS-CoV-2 regulations. In two Italy plants using one sample per month, a significant increase in cocaine consumption was observed in 2020 (Di Marcantonio et al. 2022). Obviously, there was a shift from methamphetamine to cocaine, but whether this happened due to the measures against SARS-CoV-2 infections cannot be clarified here (Figs. 4 and 5). In Reykjavik, 24-h composite wastewater samples of a week were examined, for example, in June 2020. The estimated amphetamine and methamphetamine use showed signs of increasing from 2017 to 2020, cocaine use increased from 2017 to 2019 and decreased in 2020 during the COVID-19 pandemic (Love et al. 2022). Very different effects of the COVID-19 pandemic on drug use can be observed across Europe (Been et al. 2021). On the one hand, this has to do with national and local peculiarities and differences. On the other hand, the data situation is different, which limited comparability.

MDMA loads were lower during the initial lockdown phase, probably party- and event-related consumption was reduced. In particular, on the weekends of the first lockdown, the measured MDMA quantities were significantly lower than in later periods. In contrast, other drugs did not show this pattern. Despite more severe restriction measures during Lockdown2a than Lockdown 2b, the drug consumption differed only slightly. The data from Post Lockdown2 shows slight increases in MDMA and little lower amounts of methamphetamine, which is in line with the trend for the entire SARS-CoV-2 period. The reasons for the slight increase in amphetamine and the slight decrease in cocaine can only be speculated here. Little higher loads of cotinine were found in the first phase of lockdown; otherwise, nicotine consumption does not change.

The study is limited by the strong fluctuations in the measured values in small systems and the small number of samples at the beginning of the SARS-CoV2 pandemic.

### Study part 2—establishment of marker substances

In the present study, the beta adrenoceptor antagonist metoprolol and the nicotine metabolite cotinine were found to be suitable marker substances for the characterization of wastewater. The smallest coefficient of variation was found for these substances in the specified period (Table 5). The search for suitable marker substances in the wastewater yielded various findings. The largest amount was found from gabapentin. This can be explained by the frequent prescription in Germany (Ludwig et al. 2021), the relatively high daily defined dose of 1.8 g (Federal Institute for Drugs and Medical Devices, Germany), and the fact that gabapentin is excreted largely as unchanged compound (Thurlow et al. 1996). However, there are relatively large fluctuations in the amounts eliminated, both per site and between sites. Comparable results have been found for carbamazepine, which represented another pharmaceutical substance with frequent prescription in Germany and high daily defined dose of 1.0 g (Ludwig et al. 2021) (Federal Institute for Drugs and Medical Devices, Germany). Prescription data of the statutory health insurance AOK Plus, which represent around 41% of outpatients in Free State of Saxony, Germany (Timpel et al. 2016), showed consistently high prescriptions of gabapentin and carbamazepine in catchments of investigated WWTPs in 2020 and 2021 (unpublished prescription data in cooperation with U. Maywald, AOK Plus, Dresden, Germany).

The largest deviations in marker concentrations related to the location were at the WWTP MLHC. The high proportion of hospital wastewater in this treatment plant might contribute to these variations. The high fluctuations in the values observed in the small WWTP Elsterberg are striking and unfavorable for comparative considerations. In all other WWTPs, the excreted amounts of metoprolol showed the smallest scatter. The cotinine values determined as part of the investigations of nicotine consumption in this study also showed relatively constant values. Tobacco smoking is still common in Germany and the regulations against SARS-CoV-2 infections seemingly had only a negligible impact on consumption. In contrast to Germany, fewer tobacco compounds were found in wastewater in Athens at the beginning of the COVID-19 pandemic (Alygizakis et al. 2021). A comparison of the average amounts of cotinine and metoprolol excreted in the wastewater suggests that both substances are suitable as marker substances in Germany. Discharge of hospital sewage played no discernable role in cotinine excretion. The comparatively low values in the small WWTPs, often receiving wastewater from rural catchments, are striking. Demographic factors might contribute to this observation, since a larger proportion of older people live in rural areas. Finally, the proportion of human excretions in the wastewater can therefore be estimated from the concentrations of metoprolol and cotinine. Data in Table 5 (part 1 of the study), clearly confirm that the beta adrenoceptor antagonist metoprolol and the nicotine metabolite cotinine were excreted in constant amounts and are well suited as marker substances.

These two marker substances are useful when the wastewater cannot be characterized in terms of flow rate and inhabitants in the catchment area, for example, when sewer sub-networks were sampled. Other substances can be related to these markers, for example, to assess whether drug or substance abuse changes, i.e., varies within the week or between different phases of the lockdown.

The amounts of metoprolol and cotinine excreted differ between the sites, so the use of marker substances cannot be transferred from one WWTP to another, which limits the study. In the case of special dischargers such as hospitals or regarding very small catchment areas, the results of wastewater analysis are more difficult to interpret. The main results of the study are summarized in Table 6.

**Table 6:** Most important results

Wastewater-based epidemiology is suitable for showing changes in drug use during the COVID-19 lockdown	Change in illicit drug use was present at the beginning of the SARS-CoV-2 crisis	In Nuremberg, significantly lower cocaine/benzoylecgonine content and less MDMA in the first phase of the lockdown were observed
		In Dresden and Chemnitz, methamphetamine levels increased during the lockdown1, but decreased from summer 2020 on
	Thereafter from mid-2020, no obvious effect was detected	At all times and in all WWTPs, increase of consumption of party drugs on weekends was present
		The absolute highest amounts of drugs were found at the New Year’s Eve despite comprehensive social restrictions
Metoprolol and cotinine are excreted at constant loads throughout the seasons and epidemic phases	Substances which were excreted in constant amounts can be used as markers for the waste water	Marker substances are useful when flow rate and number of inhabitants in the catchment area are unknown. Other substances can be related to these markers

## Conclusion

Wastewater-based epidemiology is suitable for showing changes in drug use during the COVID-19 lockdown. With regard to consum of illicit drugs, a distinction was made between two aspects: the absolute amount of substance and its distribution over the week. Cocaine and especially MDMA (Ecstasy) are recreational drugs; they are increasingly consumed at weekends. In Nuremberg, these wastewaters have a significantly lower cocaine/benzoylecgonine content and also less MDMA in the first phase of the lockdown in comparison with later time periods. At all other times and in all WWTPs examined, the typical increase of consumption of party drugs on weekends was found. In Dresden and to a lesser extent in Chemnitz, it was observed that methamphetamine levels increased during the lockdown1, but decreased from summer 2020 on. The absolute highest amounts of drugs were found at the New Year’s Eve despite comprehensive social restrictions. Overall, the results suggest that drug consumption changed in the observed WWTPs at the beginning of the SARS-CoV-2 pandemics but later restrictions had lesser effects on consumption behavior.

This study also showed that both the beta adrenoceptor antagonist metoprolol and the nicotine metabolite cotinine are excreted at constant loads throughout the seasons and epidemic phases. Hence, they are suitable as marker substances for the analysis of wastewater from a pharmacological point of view. Inhabitant specific loads of these markers differed among the sites. So, a local adjustment of expected consumption levels seems necessary. Pharmacological markers provide reliable site specific validation, of sample integrity or when the amount of wastewater at the sampling point is unknown.

## Supplementary Information

Below is the link to the electronic supplementary material.

Supplementary file 1 (Docx 15.8 KB)

## Data Availability

Not applicable.

## Abbreviations

EMCDDA: European Monitoring Center for Drugs and Drug Addiction
MDMA: 3,4-Methylenedioxymethamphetamine
PE: Population equivalents
SCORE: Sewage Analysis Core Group Europe
WBE: Wastewater-based epidemiology
WWTP: Wastewater treatment plants
