# Supplementary material for: Drug consumption in German cities and municipalities during the COVID-19 lockdown: a wastewater analysis
Source: Naunyn Schmiedebergs Arch Pharmacol. 2023 Jan 12;396(5):1061–74. doi: 10.1007/s00210-022-02377-2 (PMC9836342; doi:10.1007/s00210-022-02377-2)
Supplement: Supplementary file 1 — (Docx 15.8 KB) [file 210_2022_2377_MOESM1_ESM.docx]

Table 7: Number of samples per sewage treatment plant and per time period

| Period |  | 1 April – 30 April 2020 | 1 May – 15 June 2020 | 16 June – 31 Oct. 2020 | 1 Nov. – 15 Dec. 2020 | 16 Dec. – 28 Feb. 2021 | 1 March – 31 May 2021 | 1 June – 22 Nov. 2021 | 23 Nov. 2021-2 Jan. 2022 |
| --- | --- | --- | --- | --- | --- | --- | --- | --- | --- |
| WWTP |  | Lockdown 1a (LD1a) | Lockdown 1b (LD1b) | post Lockdown 1 (pLD1) | Lockdown light 2 (LDli2) | Lockdown 2a (LD2a) | Lockdown 2b (LD2b) | post Lockdown 2 (pLD2) | Lockdown light 3 (LDli3) |
| Annaberg-Buchholz | 123 | 0 | 0 | 0 | 3 | 10 | 36 | 64 | 10 |
| Chemnitz | 151 | 8 | 9 | 0 | 3 | 10 | 34 | 66 | 21 |
| Dresden | 226 | 1 | 16 | 18 | 11 | 43 | 50 | 67 | 20 |
| Hamburg Nord | 25 | 0 | 13 | 12 | 0 | 0 | 0 | 0 | 0 |
| Hamburg Süd | 26 | 0 | 13 | 13 | 0 | 0 | 0 | 0 | 0 |
| Nürnberg | 32 | 14 | 13 | 5 | 0 | 0 | 0 | 0 | 0 |
| Magdeburg | 42 | 13 | 16 | 13 | 0 | 0 | 0 | 0 | 0 |
| Illingen Wustweiler | 20 | 0 | 10 | 10 | 0 | 0 | 0 | 0 | 0 |
| Saarbrücken Brebach | 20 | 0 | 10 | 10 | 0 | 0 | 0 | 0 | 0 |
| Saarbrücken Burbach | 19 | 0 | 9 | 10 | 0 | 0 | 0 | 0 | 0 |
| Saarlouis | 20 | 0 | 11 | 9 | 0 | 0 | 0 | 0 | 0 |
| Plauen | 112 | 0 | 0 | 0 | 0 | 0 | 39 | 61 | 12 |
| MLHC | 3 | 0 | 0 | 0 | 3 | 0 | 0 | 0 | 0 |
| Elsterberg | 18 | 0 | 0 | 0 | 0 | 0 | 18 | 0 | 0 |
| MRK | 23 | 0 | 0 | 0 | 0 | 0 | 23 | 0 | 0 |
| total number | 860 | 36 | 120 | 100 | 20 | 63 | 200 | 258 | 63 |

Table 8: Excreted amount of illegal drugs in raw wastewater of four WWTPs in the federal state Saarland [mg/day /1,000 inhabitants]

|  | | | | | | | | | | | | | | | | |  | | | | | | | | | | | | | |
| --- | --- | --- | --- | --- | --- | --- | --- | --- | --- | --- | --- | --- | --- | --- | --- | --- | --- | --- | --- | --- | --- | --- | --- | --- | --- | --- | --- | --- | --- | --- |
|  | WWTP Brebach | | |  | |  | | WWTP Burbach | | |  | |  | | WWTP Wustweiler | | | |  | |  | | WWTP Saarlouis | | |  | |  | |  |
|  | N | Amphetamine | Benzoylecgonine | | MDMA | | N | | Amphetamine | Benzoylecgonine | | MDMA | | N | | Amphetamine | | Benzoylecgonine | | MDMA | | N | | Amphetamine | Benzoylecgonine | | MDMA | |  |  |
| pre Lockdown | 7 | 274 | 66 | | 7,4 | | 7 | | 386 | 243 | | 22,5 | | 0 | | no data | | no data | | no data | | 0 | | no data | no data | | no data | |  |  |
| Lockdown1a | 10 | 199 | 87 | | 18,3 | | 9 | | 356 | 216 | | 26,3 | | 10 | | 209 | | 40,1 | | 13,3 | | 11 | | 291 | 120 | | 27,6 | |  |  |
| post Lockdown | 10 | 213 | 89 | | 18,3 | | 10 | | 427 | 228 | | 33,9 | | 10 | | 227 | | 35,0 | | 12,6 | | 9 | | 323 | 114 | | 22,4 | |  |  |
